# Surgical strategies for total colonic aganglionosis: a retrospective comparative study

**DOI:** 10.3389/fsurg.2026.1846434

**Published:** 2026-09-28

**Authors:** Ahmed Arafa, Abdelhalem Showkat Mohamed, Ahmed E. Arafat, Belal Elezaby, Mahmoud Marei Marei, Seham Anwar, Ahmed Azzam, Ahmed S. Ragab

**Affiliations:** 1 Pediatric Surgery Department, Faculty of Medicine, Cairo University, Cairo, Egypt; 2 Department of General Surgery, Faculty of Medicine, Minia University, Minia, Egypt; 3 Pediatric Surgery Department, Faculty of Medicine, Beni Suef University, Beni Suef, Egypt; 4 Pediatric Surgery Department, Faculty of Medicine, Port Said University, Port Said, Egypt

**Keywords:** different surgical techniques, Duhamel's, ileoanal, laparoscope, TCA, Swenson

## Abstract

**Aim:**

The study aimed to evaluate different surgical techniques in total colonic aganglionosis cases.

**Methods:**

Twenty patients were included in this study. A total colectomy, except for the lower rectum in cases of Duhamel's. The average age for an ileal pull-through in ileostomized cases was 1 year; ileostomy closure was performed after 3 years. In eight non-ileostomized cases, surgery was performed at 2–3 years of age. Group A comprised 10 cases that underwent direct ileoanal pull-through (seven open and three laparoscopic), while Group B comprised 10 cases that underwent Duhamel's procedure (six laparoscopic and four open). Operative details and postoperative outcomes were recorded for the entire cohort. Comparative statistical analysis was performed on patients with complete follow-up data (*n* = 20).

**Results:**

All patients were followed up for 5 years after surgery. Postoperative complications included fecal incontinence, perianal dermatitis, electrolyte disturbances, enterocolitis, and constipation. Fecal incontinence and electrolyte disturbances were more frequently observed after straight ileoanal pull-through, whereas enterocolitis and constipation were more common after Duhamel's procedure. No anastomotic leakage was reported. Comparative analysis showed no statistically significant differences between the two techniques.

**Conclusion:**

No clear superiority was demonstrated between straight ileoanal pull-through and Duhamel's procedure in patients with total colonic aganglionosis. Each technique was associated with a distinct pattern of complications, and larger studies are needed to better define optimal surgical management.

## Background

Total colonic aganglionosis (TCA), defined as aganglionosis extending from the anus up to 30–50 cm proximal to the ileocecal valve (1, 2), is a rare variant of Hirschsprung’s disease, occurring in approximately 3%–10% of all cases (3).

The goal of surgical therapy is to remove the aganglionic intestine. Numerous surgical methods including straight ileoanal anastomosis and Duhamel's procedure have been documented over time (4–8). A 2009 meta-analysis, however, did not show that any of them produced better outcomes (9).

Recent advances in laparoscopy offer better visualization, cosmesis, and faster recovery, but long-term outcomes remain under evaluation (10, 11).

While several historical series have compared surgical techniques for TCA, contemporary comparative data remains scarce due to the extreme rarity of the condition. Furthermore, many existing studies are constrained by brief postoperative windows that fail to capture the evolving landscape of long-term functional outcomes. This study addresses these gaps by evaluating a dedicated cohort of TCA patients undergoing surgical techniques (e.g., laparoscopic Duhamel’s vs. Swenson’s procedures). By analyzing extended long-term follow-up data alongside a standardized assessment of late-stage complications, this paper provides novel real-world insights into the durability, procedural safety, and long-term clinical trajectories associated with each approach.

## Methods

Twenty cases of TCA-diagnosed individuals participated in this retrospective investigation. Among these,12 cases involved ileostomies (six in the Duhamel group, six in the ileoanal pull-through group) performed in the neonatal period due to intractable intestinal obstruction or recurrent enterocolitis. The average age for an ileal pull-through in ileostomized cases was 1 year, with ileostomy closure performed after 3 years of age. In eight non-ileostomized cases, surgery was performed at 2–3 years of age. This retrospective cohort study was conducted across four tertiary pediatric surgery units (Cairo, Beni Suef, Minia, and Port Said University Hospitals) from 2018 to 2022. Patients were managed at the participating institutions, and some cases were referred between centers according to the availability of expertise. Surgical procedures were performed by experienced pediatric surgeons at each center.

### Statistical analysis

Statistical analyses were performed using IBM SPSS Statistics version 26.0 (IBM Corp., Armonk, NY, USA). Continuous variables were assessed for normality using the Shapiro–Wilk test; given the small cohort size (*n* = 20), continuous data are expressed as medians and IQR and compared using the Mann -Whitney U-test. Categorical variables are presented as frequencies/percentages and compared using the Chi-square (*χ*²) test, with Fisher's exact test used when expected cell frequency was <5. Two-sided *p* < 0.05 was considered significant, with 95% CIs reported for primary outcomes. A multivariate regression model was not performed given the sample size.

To maximize statistical rigor despite the limited sample power, two-sided *p*-values are reported alongside 95% confidence intervals (95% CI) for primary outcome rates. Statistical significance was defined as *p* < 0.05. Due to the constrained sample size, a multivariate regression model was not feasible to avoid statistical overfitting.

Patients were strictly included in this study if they met the following criteria:

- A definitive, histologically confirmed diagnosis of total colonic aganglionosis, confirmed via full-thickness rectal biopsy or intraoperative mapping.
- Complete medical records and continuous follow-up for a minimum of 5 years postoperatively.
- Definitive surgical reconstruction performed at our affiliated university hospitals using either the straight ileoanal pull-through or Duhamel’s procedure.

Patients with total intestinal aganglionosis extending beyond the ileum or with major associated cardiac anomalies were excluded. However, patients with total colonic aganglionosis with limited ileal extension were included, provided that the aganglionic segment did not exceed 25 cm proximal to the ileocecal valve. Patients with aganglionosis extending beyond this level (total intestinal aganglionosis) were excluded. All patients were screened for additional anomalies, including genitourinary, neurological, and chromosomal abnormalities; no significant anomalies were identified.

The choice of surgical procedure (straight ileoanal pull-through vs. Duhamel's procedure) was based on surgeon preference, intraoperative findings, and patient-related factors, including bowel caliber, nutritional status, and presence of enterocolitis. This approach reflects real-world practice in a retrospective setting.

Allocation to either Duhamel’s or Swenson’s technique was non-randomized and dictated by specific clinical, anatomical, and chronological factors rather than baseline disease severity.

Duhamel’s procedure was preferentially utilized in cases presenting with significant pelvic disparity, a highly dilated proximal bowel segment where a wide side-to-side anastomosis was technically advantageous, or in patients managed by senior surgeons specialized in this approach.

Conversely, Swenson’s procedure was favored in cases with minimal pelvic soft-tissue inflammation, a well-matched diameter between the ganglionic donor bowel and the anal canal, or during the later period of the study as institutional expertise shifted toward total endorectal dissection to minimize posterior rectal pouch complications. All procedures were executed or directly supervised by attending pediatric surgeons to ensure technical consistency.

These instances were categorized into two groups:

- Group A: Direct ileoanal pull-through was performed in 10 cases (seven open and three laparoscopic).
- Group B: Duhamel's procedure was performed in the other 10 cases (six laparoscopic and four open).

Clinical suspicion of TCA was based on delayed passage of meconium, recurrent intestinal obstruction, abdominal distension, or recurrent enterocolitis. Diagnostic evaluation included contrast enema studies and full-thickness rectal biopsies performed under general anesthesia. Distal loopograms were obtained prior to ileostomy closure to detect fistulae or strictures (Figure 1). Intraoperative frozen-section biopsies were performed in an ascending manner to confirm the proximal extent of aganglionosis.

**Figure 1:**
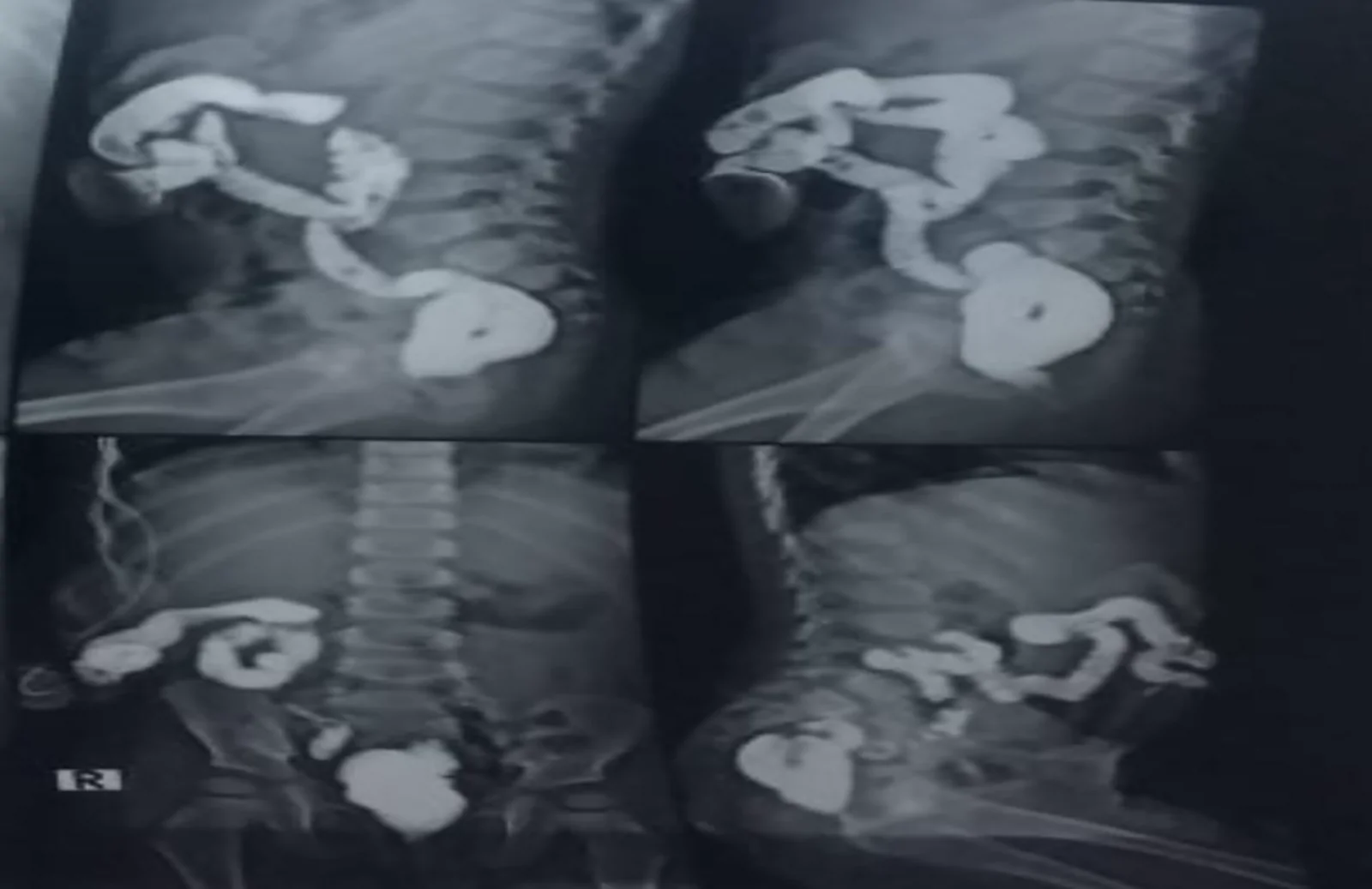
Distal loopogram obtained before ileostomy closure in a patient who underwent laparoscopic Duhamel reconstruction for TCA. The study demonstrates a patent colorectal anastomosis without evidence of anastomotic leakage, fistula formation, or anastomotic stricture. This image illustrates the routine radiological assessment performed before stoma closure.

Patients who had not undergone ileostomy were managed conservatively prior to surgery with regular rectal irrigations, nutritional optimization, and close clinical monitoring. Non-ileostomized patients who developed enterocolitis in early life responded to conservative management, including rectal irrigation, bowel rest (NPO), and antibiotics.

### Operative procedure

The operative procedure comprised two components: a transanal component and an intra-abdominal component.

#### Laparoscopic (intra-abdominal) component

- Pneumoperitoneum was maintained at a flow rate of 2 L/min and a pressure of 10 mmHg. Four ports were used: a camera port at the umbilicus, one port two fingerbreadths below the xiphisternum, one port in the right iliac fossa for dissection of the left colon, and one port in the left iliac fossa for dissection of the right colon.
- Laparoscopic colonic biopsies were obtained for fresh frozen section analysis in an ascending manner to confirm that the entire colon was aganglionic prior to excision (Figure 2). Laparoscopic devascularization of the entire colon was then performed (Figure 3).
- In ileoanal pull-through cases, the entire circumference of the aganglionic rectum was mobilized. In Duhamel cases, a retrorectal space was created under guidance via digital rectal examination, approximately 1 cm above the dentate line. The aganglionic rectum was divided as low as possible to leave a short rectal stump. In older children, a 55-mm laparoscopic linear gastrointestinal (GI) stapler was used to transect the distal rectum. In younger children (<3 years), a Pfannenstiel incision was made to allow distal rectal dissection using a 75 mm linear GI stapler.
- Subsequently, the ileal segment was pulled through and positioned behind the rectal remnant.
- In open cases, the same steps were performed via a midline abdominal incision.

#### Transanal part in open cases and laparoscopic cases

- A rectal incision was made 1 cm above the dentate line in all cases. In ileoanal pull-through cases, a full circular incision was performed. In Duhamel cases, a lower semicircular incision was made from 3 o’clock to 9 o'clock. The ileal segment was then pulled through, and the anterior wall of the ileum was anastomosed to the common rectal wall using a 75-mm linear GI stapler, followed by completion of the ileoanal anastomosis with 4/0 Vicryl sutures. Care was taken to ensure the absence of colonic twisting or intra-abdominal hemorrhage.

#### Postoperative anal dilatation

Anal dilatation was initiated 3 weeks postoperatively using Hegar dilators. Dilatation was performed twice weekly for the first 6 months and then once weekly for an additional 6 months.

**Figure 2:**
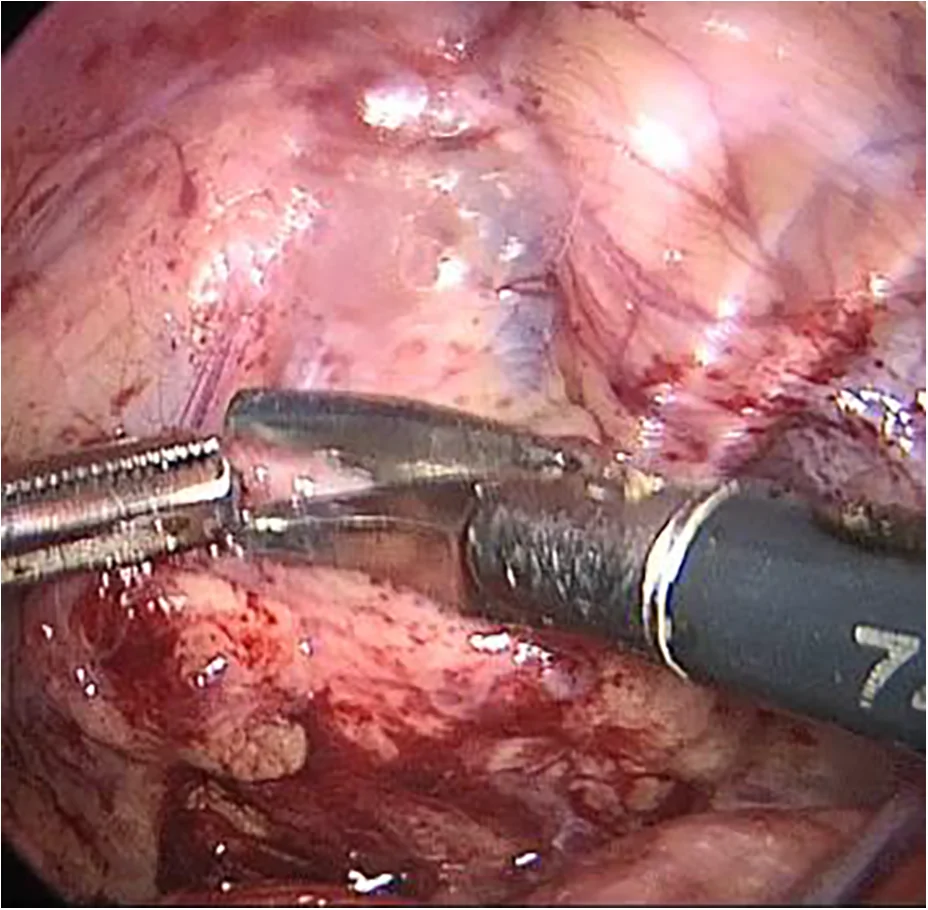
Intraoperative laparoscopic full-thickness bowel biopsy performed for frozen-section analysis during surgery for TCA. Sequential biopsies were obtained in an ascending fashion to accurately identify the proximal level of ganglionated bowel before definitive reconstruction.

**Figure 3:**
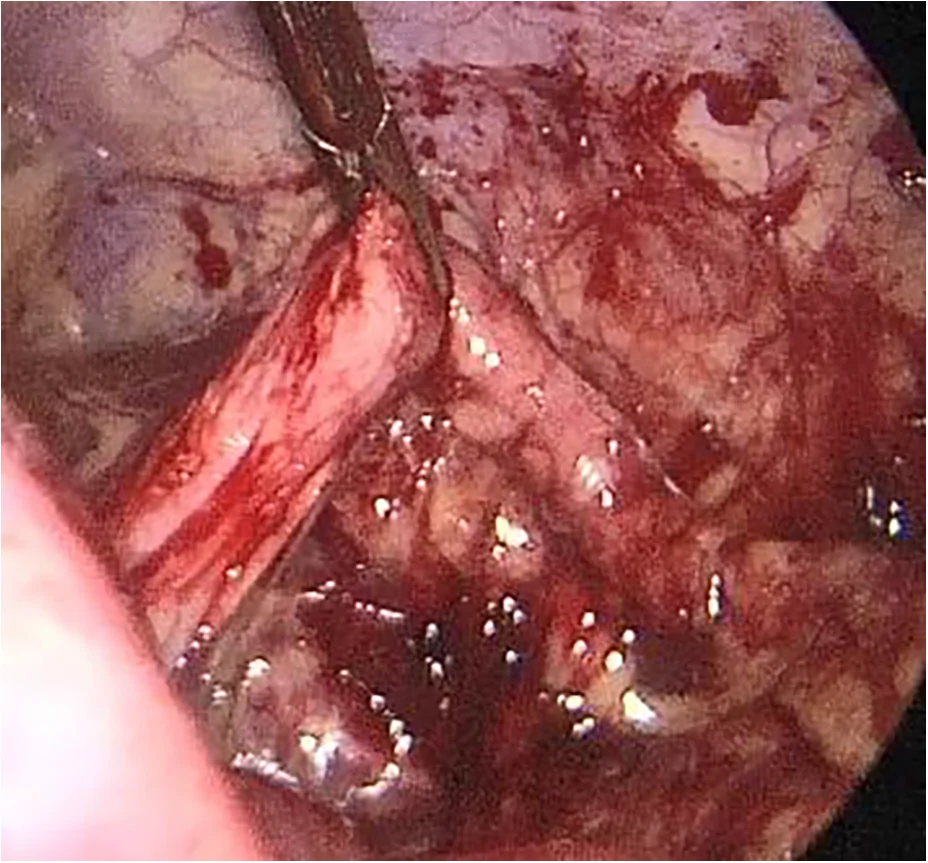
Laparoscopic devascularization of the aganglionic colon during definitive surgical treatment for TCA. Careful vascular control facilitates safe mobilization before pull-through reconstruction.

#### Postoperative complications and outcome definitions

To ensure objective and reproducible comparative tracking, clinical outcomes were strictly defined according to established international criteria:

- Postoperative constipation/fecal retention: This was defined according to the adapted Rome IV criteria for pediatric functional constipation. It was specifically characterized by fewer than two bowel movements per week, a clinical history of painful or hard bowel movements, excessive straining, or a palpable fecal mass during physical examination, requiring regular dietary adjustments, laxative therapy, or mechanical bowel irrigation for more than 3 consecutive months.
- Fecal incontinence/soilage: This was evaluated in children who achieved the developmental age of fecal continence (≥3 years old). Continuous or involuntary passage of stool into clothing was classified as fecal incontinence. Recurrent, small-volume staining of underwear during flatus or between scheduled bowel movements was explicitly defined as intermittent soilage.
- Electrolyte disturbance: This was defined as any documented post-pull-through biochemical imbalance requiring medical intervention or hospitalization. This includes acute hyponatremia (serum sodium <135 mmol/L) and/or dehydration secondary to high-output stomal or ileoanal fluid loss, necessitating intravenous fluid resuscitation and electrolyte replacement therapy.

#### Functional outcome assessment

Long-term functional outcomes regarding bowel habit and continence were evaluated during late follow-up clinical visits. While a formalized, cumulative Rintala Bowel Function Score was not systematically computed retroactively in all baseline charts, functional tracking strictly adhered to objective clinical parameters defined by consensus scoring systems.

Continuous fecal continence was categorized into three distinct clinical trajectories: (1) good/normal continence (voluntary control, no soilage, clean underwear); (2) intermittent soilage (occasional smearing during episodes of loose stools or physical exertion, not requiring social isolation); and (3) fecal incontinence/poor control (continuous involuntary loss of stool or continuous utilization of diapers beyond the expected age of developmental continence). Associated long-term sequelae, including chronic constipation and the frequency of enterocolitis episodes, were similarly tabulated.

## Results

Operative characteristics are summarized in Table 1., The outcomes of different surgical techniques during the 5-year follow-up period are presented in Table 2.

**Table 1:** Operative details among groups.

Operative details	Laparoscopic ileoanal (three cases)	Laparoscopic Duhamel (six cases)	Open ileoanal (seven cases)	Open Duhamel (four cases)
Mean duration of operation (h)	3	4	2	3
Blood transfusion	1/3	2/6	1/7	1/4
Mean hospital stay (days)	3	3	5	6

**Table 2:** Outcomes of different surgical techniques.

Outcome	Laparoscopic ileoanal	Laparoscopic Duhamel	Open ileoanal	Open Duhamel
Leakage	No	No	No	No
Stenosis	2/3	no	2/7	no
Perianal dermatitis	3/3	1/6	3/7	1/4
Fecal incontinence	3/3	1/6	3/7	1/4
Electrolyte disturbance	2/3	1/6	4/7	1/4
Enterocolitis	1/3	3/6	2/7	2/4
Constipation	1/3	2/ 6	2/7	2/4

Four instances of stenosis in Group A responded to routine anal dilatation, which was started 3 weeks postoperatively, twice per week for 6 months, then once per week for another 6 months. One of them did not respond to regular dilatation, and one patient required reoperation to excise the ischemic ileal part and redo the ileal pull-through before ileostomy closure.

Fecal incontinence and perianal dermatitis occurred, particularly in the first year after surgery, especially in cases of ileal pull-through. Statistical analysis for fecal incontinence outcome among groups is shown in Table 3. Postoperative fecal continence was clinically evaluated after a 5-year follow-up period.

**Table 3:** Comparison of fecal incontinence between groups.

Group	Fecal incontinence, *n* (%)	No incontinence, *n* (%)	Total
Ileoanal group	6 (60)	4 (40)	10
Duhamel group	2 (20)	8 (80)	10
Total	8 (40)	12 (60)	20

Statistical test: Fisher's exact test.

*p*-Value = 0.170 (not statistically significant).

Fecal continence was assessed clinically and categorized as complete incontinence (daily uncontrolled stool passage requiring diaper use), partial incontinence (intermittent soiling), or acceptable continence (voluntary bowel movements without social impairment) (Table 4). Six cases complaining of complete incontinence improved with medical treatment: Imodium (loperamide) 1 mg orally three times a day. Frequent dressing with antibiotic cream was used in cases of dermatitis.

**Table 4:** Postoperative fecal continence by surgical technique.

Continence status	Laparoscopic ileoanal (*n* = 3)	Laparoscopic Duhamel (*n* = 6)	Open ileoanal (*n* = 7)	Open Duhamel (*n* = 4)
Complete incontinence	1/3	0/6	3/7	1/4
Partial incontinence	1/3	1/6	1/7	0/4
Acceptable continence	1/3	5/6	3/7	3/4

Electrolyte imbalance occurred, especially in cases of direct ileoanal pull-through. Electrolyte disturbance was defined as documented hyponatremia (serum sodium <130 mmol/L) and/or hypokalemia (serum potassium <3.0 mmol/L), associated with clinical signs of dehydration (reduced urine output, dry mucous membranes, or weight loss) and requiring hospital admission for intravenous fluid and electrolyte replacement. All episodes occurred during the early postoperative period (within the first 3 months) and resolved with conservative management. Statistical analysis for electrolyte disturbance is shown in Table 5.

**Table 5:** Comparison of electrolyte disturbance between groups.

Group	Electrolyte disturbance, *n* (%)	No disturbance, *n* (%)	Total
Ileoanal group	6 (60)	4 (40)	10
Duhamel group	2 (20)	8 (80)	10
Total	8 (40)	12 (60)	20

Statistical test: Fisher's exact test.

*p*-Value = 0.170 (not statistically significant).

Six cases were admitted to intermediate care, requiring correction of dehydration and electrolyte imbalances, with frequent hypokalemia noted in four cases.

In contrast, patients in the Duhamel group experienced a higher incidence of postoperative enterocolitis and constipation. Constipation was defined as reduced stool frequency requiring regular use of laxatives or rectal irrigation, with or without radiological evidence of fecal loading. Enterocolitis was diagnosed based on clinical presentation (abdominal distension, diarrhea, fever), supported by radiological findings and laboratory evidence of infection, and required hospital admission for conservative management in the form of intravenous antibiotics and frequent rectal irrigations.

Statistical analysis for the enterocolitis group is provided in Table 6.

**Table 6:** Comparison of enterocolitis between groups.

Group	Enterocolitis, *n* (%)	No enterocolitis, *n* (%)	Total
Ileoanal group	3 (30)	7 (70)	10
Duhamel group	5 (50)	5 (50)	10
Total	8 (40)	12 (60)	20

Statistical test: Fisher's exact test.

*p*-Value = 0.650 (not statistically significant).

These patients required hospital admission for frequent rectal irrigation and IV antibiotics. No anastomotic leak occurred in any of the groups.

Fecal continence was assessed clinically and categorized as complete incontinence (daily uncontrolled stool passage requiring diaper use), partial incontinence (intermittent soiling), or acceptable continence (voluntary bowel movements without social impairment).

Our interdisciplinary team of pediatric surgeons, gastroenterologists, and nutritional specialists saw all patients frequently because ileal resection combined with colectomy may result in rapid bowel transit and significant postoperative malabsorption of nutrients. For this reason, close follow-up was necessary, including monitoring of electrolytes, vitamins, and growth.

In statistical analysis, we examined two broad technique categories (straight ileoanal pull-through vs. Duhamel's).

For comparisons between categorical variables across groups, Fisher's exact test was used instead of the Chi-square test due to the small sample size (*n* = 20), where many expected cell counts were below 5. A *p*-value <0.05 was considered statistically significant.

- Fecal incontinence: Fecal incontinence was reported in 60% of patients with direct ileoanal pull-through compared to only 20% in Duhamel’s procedure. However, this difference was not statistically significant (*p* = 0.170, Fisher's exact test).
- Electrolyte disturbance: Occurred in 60% of cases in the ileoanal group vs. 20% in the Duhamel group, with no statistically significant difference (*p* = 0.170).
- Enterocolitis: Reported in 30% of the ileoanal group and 50% of the Duhamel group. Again, the difference did not reach statistical significance (*p* = 0.650).
- None of the clinical outcomes (incontinence, electrolyte disturbance, or enterocolitis) showed statistically significant differences between groups.
- Although qualitative differences were observed in complication patterns between the surgical techniques, these were not supported by statistical evidence and should not be overstated.
- Raw SPSS output tables were excluded; instead, key comparisons were summarized directly in the text with percentages and *p*-values clearly stated.
- These findings highlight the need for studies with larger sample sizes to better evaluate surgical outcomes in total colonic aganglionosis.

We found no statistically significant differences in complication rates between the two techniques. No obvious superiority was found in either of these surgical techniques. Fecal incontinence and electrolyte disturbance were more frequently detected in direct ileal pull-through, while enterocolitis was more common with colonic use as a rectal pouch.

Among cases with ileostomies, we observed seven cases of prolapsing ileostomy (Figure 4). These patients were managed conservatively, and we had no leakage after ileostomy closure.

**Figure 4:**
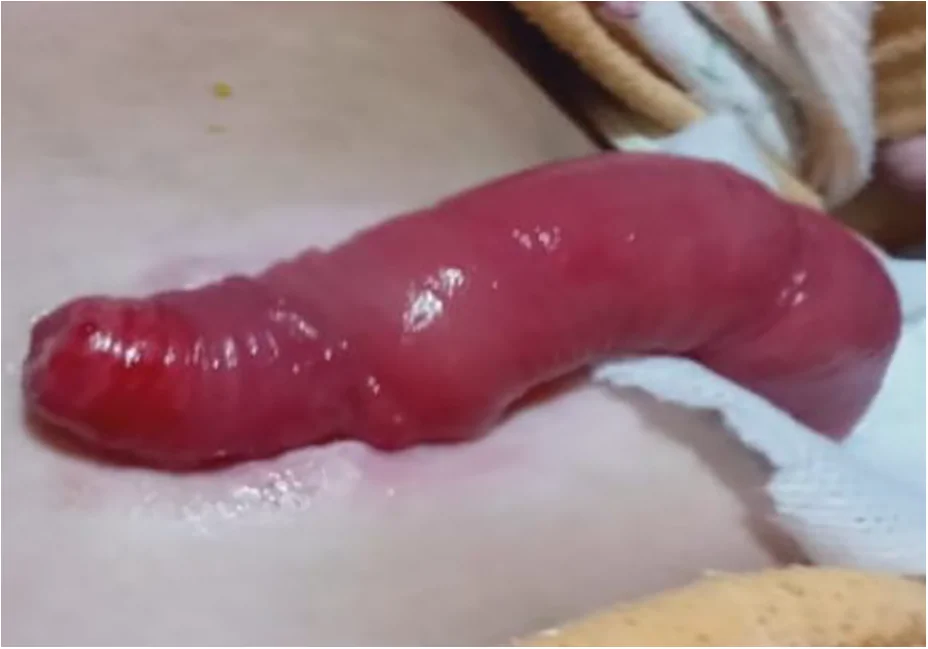
Prolapsed ileostomy observed during postoperative follow-up. The prolapse was managed conservatively without surgical revision.

Statistical analyses were performed using SPSS version 26.0 (IBM Corp., Armonk, NY, USA). Continuous variables were assessed for normality using the Shapiro–Wilk test. Given the small cohort size (*n* = 20) inherent to the rarity of TCA, continuous data are expressed as medians and IQR and were compared using the non-parametric Mann–Whitney *U*-test. Categorical variables are presented as frequencies and percentages and were compared using Fisher's exact test.

To maximize statistical rigor despite the limited sample power, two-sided *p*-values are reported alongside 95% CI for primary outcome rates. Statistical significance was defined as *p* < 0.05. Due to the constrained sample size, a multivariate regression model was not feasible to avoid statistical overfitting.

There was no statistically significant difference between the ileoanal and Duhamel groups regarding constipation [odds ratio (OR) 0.64, 95% CI 0.10–4.10; *p* = 1.000], fecal incontinence (OR 6.00, 95% CI 0.81–44.35; *p* = 0.170), or electrolyte disturbance (OR 6.00, 95% CI 0.81–44.35; *p* = 0.170) (Table 7).

**Table 7:** Comparative outcomes (constipation, fecal incontinence, electrolyte disturbance) between the ileoanal pull-through and Duhamel's procedure groups, with odds ratios, 95% confidence intervals, and Fisher's exact *p*-values.

Outcome	Ileoanal (A) *n* = 10	Duhamel (B) *n* = 10	Odds ratio (95% CI)	Fisher's exact *p*
Constipation	3 (30%)	4 (40%)	**0.64** (0.10–4.10)	**1**.**000**
Fecal incontinence	6 (60%)	2 (20%)	**6.00** (0.81–44.35)	**0**.**170**
Electrolyte disturbance	6 (60%)	2 (20%)	**6.00** (0.81–44.35)	**0**.**170**

## Discussion

Our main challenges after total colectomy were lifestyle limitations due to high gastrointestinal motility and fecal incontinence. When the entire colon is removed, intestinal contents flow more rapidly, leading to less fluid retention. We had two groups: Group A comprised 10 cases that underwent direct ileoanal pull-through (seven open and three laparoscopic) and Group B comprised 10 cases that underwent Duhamel's procedure (six laparoscopic and four open Duhamel's).

The retrospective nature of this study inherently introduces potential selection bias regarding surgical procedure allocation. The choice between Duhamel’s and Swenson’s techniques was non-randomized and heavily influenced by surgeon preference, historical institutional eras, and intraoperative anatomical constraints. For instance, Duhamel's procedure was preferred in cases with proximal bowel dilatation, while ileo-anal pullthrough was performed for cases with good pelvic anatomy, no adhesions, and a small-diameter distal colon. However, to mitigate the impact of this bias on our comparative outcomes, baseline patient demographics and initial clinical presentation severity were comparable between both groups. While this bias is difficult to eliminate entirely in retrospective series of rare conditions, our data still provides essential real-world clinical insight into the long-term management of TCA.

In the context of TCA, the choice of definitive reconstruction remains highly debated. While some centers lean toward a straight ileoanal pull-through, our institutional experience highlights critical socioeconomic and clinical considerations often overlooked in Western literature. In developing countries, where immediate access to specialized emergency care can be delayed and financial constraints limit continuous supportive home care, the risk of rapid dehydration and catastrophic electrolyte disturbances poses a major threat.

Therefore, we argue that the creation or utilization of a surgical reservoir—as inherent to Duhamel’s procedure—provides a vital clinical safety net. By acting as a functional reservoir that slows intestinal transit and optimizes fluid and electrolyte absorption, it significantly reduces the incidence of severe, life-threatening dehydration episodes compared to a straight ileoanal pull-through. Consequently, our 5-year follow-up data demonstrates that in resource-limited settings, preserving a reservoir mechanism is not merely an option for functional continence but a strategic surgical decision to minimize severe metabolic morbidity.

The management of TCA presents a unique set of long-term surgical challenges compared to classic short-segment Hirschsprung's disease. Our 5-year follow-up data demonstrates that while both Duhamel’s and Swenson’s procedures achieve successful anatomic reconstruction, their long-term complication profiles stem from distinct mechanical and pathophysiological mechanisms. When contextualizing these findings within contemporary global evidence, our outcomes closely align with recent international multicenter registry studies and multi-institutional collaborative series. Modern database analyses, such as those from the ERNICA consortium, consistently indicate that TCA remains associated with substantial long-term morbidity regardless of the chosen pull-through technique.

In our cohort, enterocolitis (HAEC) remained a significant late-stage morbidity across both groups, a finding that mirrors modern multicenter reports where late-onset HAEC remains a persistent clinical hurdle. Pathophysiologically, the absence of the entire colon eliminates a massive reservoir for fluid absorption, leading to a chronically loaded, dilated small bowel highly susceptible to bacterial overgrowth and dysmotility. In the Swenson group, late HAEC episodes were often mechanically linked to minor anastomotic strictures or relative hypertonia of the anal sphincter, which creates an outflow obstruction.

Conversely, in the Duhamel group, while the creation of a colorectal pouch provided a crucial metabolic safety net against rapid electrolyte loss (a trend similarly noted in large-scale database analyses favoring Duhamel for fluid management), it inherently introduced the risk of a “blind pouch” effect. Fecal retention and incomplete emptying within the posterior rectal reservoir can lead to chronic stasis, secondary bacterial proliferation, and subsequent recurrent enterocolitis. This highlights that long-term functional success in TCA depends not only on the initial pull-through execution but also on a proactive, years-long regimen of bowel management to optimize transit and prevent reservoir dilatation. By integrating our single-center physiological observations with these contemporary multicenter cohorts, we reinforce that surgical individualization must be paired with specialized, long-term postoperative surveillance.

The mainstay of treatment for TCA is the excision of the aganglionic segment, followed by either a direct ileoanal pull-through or an ileal pull-through performed in conjunction with a remnant of aganglionic colon to reduce high stool frequency and malabsorption. Several surgical methods have been described, each with its own advantages and disadvantages (4–9).

Regarding ileoanal pull-through, Bischoff et al. (4) reported 27 cases who underwent total colectomy with straight ileoanal pull-through, all of whom received a protective ileostomy. In contrast, Cheung et al. (12) performed laparoscopic endorectal pull-through in 10 patients without a stoma. In our series, we used preoperative laparoscopic ileostomy in only 12 cases for staging repair in case of incidental intractable enterocolitis or obstruction in neonates, while Dodero et al. (13) reported total colectomy with straight ileoanal Soave endorectal pull-through in 42 cases, noting no mortality or major morbidity aside from perianal excoriation, a complication also described by Bischoff et al. (4). To minimize this, definitive pull-through can be delayed until toilet training, as high stool frequency in the early postoperative period typically improves over time.

In our ileoanal pull-through cases, definitive surgery was performed early in ileostomized patients, with ileostomy closure delayed until toilet training. Postoperative complications included perianal dermatitis, fecal incontinence, and significant electrolyte disturbances in the majority of cases, often requiring hospital admission and intermediate care. In contrast, patients who underwent Duhamel’s procedure predominantly experienced enterocolitis and constipation, mostly attributable to the residual aganglionic rectal segment. Although no statistically significant differences were identified between surgical techniques, the clinical impact of postoperative complications should be interpreted beyond their frequency alone. Electrolyte disturbances, more frequently observed after straight ileoanal pull-through, may represent more clinically significant morbidity due to their acute nature, need for hospital admission, and potential for recurrence, particularly in young infants. In contrast, other complications observed in the Duhamel group, such as constipation or enterocolitis, were generally manageable with medical treatment and did not carry the same immediate risk.

Some authors have advocated using the aganglionic colon as a reservoir during anastomosis. Yeh et al. (14) reported a modified Duhamel’s procedure in nine TCA cases, achieving normal bowel control with soiling in only two patients. Ross et al. (15) performed Martin's procedure in 12 children, reporting a 0% mortality rate and 81.8% morbidity, with longer aganglionic segments correlating with higher complication rates. Zhang et al. (16) and Xu et al. (17) performed laparoscopic Duhamel’s procedures with rectal pouches of 5 and 10 cm, respectively. In our Duhamel cases, we created a short rectal stump (5 cm) to form a pouch, aiming to reduce the risk of recurrent enterocolitis and constipation.

No clear superiority was observed between the two surgical procedures; the choice of technique can be guided by the surgeon's ability to mitigate the disadvantages associated with each approach. This is supported by Halaweish et al. (18), who reported 54 TCA cases—26 undergoing Duhamel’s and 28 undergoing Swenson’s pull-through—and found no significant differences in medical comorbidities, operative details (including age at pull-through, laparoscopic vs. open approach, length of involved small bowel, and operative time), length of hospital stay, or postoperative complications. However, unlike the multicenter study, our analysis focused on the pattern of postoperative morbidity observed during long-term follow-up. Although the overall complication rates were comparable, we observed clinically distinct complication profiles, with electrolyte disturbances and fecal incontinence occurring more frequently after straight ileoanal pull-through, whereas enterocolitis and constipation predominated after Duhamel’s procedure. These differences, while not statistically significant, may still influence surgical decision-making in daily clinical practice, particularly in centers managing patients with limited access to specialized postoperative care.

Bischoff et al. (4) reported 21 ileostomy-related complications among 10 patients, including prolapse, stricture, and inadequate stoma formation. In our series, seven ileostomized patients experienced prolapsing ileostomies, which were managed conservatively. Bischoff et al. (4) recommended fixing the bowel to the anterior abdominal wall to prevent prolapse and performing surgery for symptomatic cases. Marquez et al. (9) reported no anastomotic leaks in their series; similarly, no early major complications were observed in either group in our study. In contrast, Stenström et al. (19) reported the need for an early ileostomy in 4 of 72 cases.

The multicenter study by Stenström et al. emphasized that long-term functional outcomes remain highly variable regardless of the surgical technique employed. Our findings support this observation, suggesting that postoperative quality of life is influenced not only by the choice of reconstruction but also by long-term multidisciplinary follow-up and management of procedure-specific complications. Therefore, careful postoperative surveillance may be as important as the initial operative approach in optimizing patient outcomes.

Direct ileoanal pull-through offers anatomical simplicity but is associated with high stool frequency, fecal incontinence, perianal dermatitis, and significant electrolyte disturbances, particularly in the early postoperative period. These findings are consistent with previous reports of high-output stools and metabolic complications following straight ileoanal anastomosis (4, 5, 12). Conversely, Duhamel’s procedure provides a functional reservoir that reduces stool frequency and electrolyte imbalance. However, this advantage is counterbalanced by higher rates of enterocolitis and constipation, likely due to retained aganglionic bowel (9, 14). In our series, limiting the length of the rectal stump appeared to mitigate these complications.

Overall, our results align with previously published studies demonstrating comparable long-term outcomes between different surgical techniques, with postoperative complication profiles—rather than mortality—being the primary distinguishing factor. Surgical choice should therefore be individualized based on patient condition, surgeon experience, and available institutional resources.

This study has several limitations that warrant consideration when interpreting our findings. First, its retrospective nature inherently restricts our control over confounding variables and baseline clinical documentation. Second, although data were collected from tertiary specialized centers, the cohort may still be subject to referral bias, as these institutions typically manage more complex or late-presentation TCA cases. Third, our sample size of 20 patients yields limited statistical power, which restricts our ability to detect subtle, statistically significant differences between Duhamel’s and Swenson’s techniques, rendering our comparative results preliminary. Lastly, the relatively heterogeneous follow-up duration across the patient cohort may influence the captured rates of late-onset complications, such as long-term fecal continence development or delayed enterocolitis episodes.

Despite these limitations, this study provides valuable insight into the long-term outcomes of the two commonly used surgical techniques for this rare condition.

In conclusion, rather than a single standardized approach for TCA, we advocate for an individualized surgical strategy that factors in both anatomical constraints and local healthcare infrastructure to minimize late-stage morbidity.

## Conclusion

Based on our findings, surgical decision-making should be tailored as follows:

Duhamel’s procedure is highly recommended in developing countries and resource-limited settings. Its creation of a colorectal pouch acts as a crucial metabolic safety net, preventing rapid, life-threatening dehydration and electrolyte crises. It is also the preferred choice when there is a significant caliber mismatch between the dilated proximal small bowel and the narrow aganglionic rectal stump.

Swenson’s procedure remains an excellent, more anatomic straight pull-through that avoids the risks of a “blind pouch” and fecal stasis. However, it should be reserved for institutions with dedicated, long-term specialized bowel management programs capable of closely monitoring and managing late anastomotic strictures and outflow obstruction.

No clear superiority was observed between the surgical techniques. Fecal incontinence and electrolyte disturbances were more frequently observed following direct ileoanal pull-through, whereas enterocolitis was more common when a colonic segment was used as a rectal pouch (Duhamel’s procedure). Larger series with extended follow-up are needed to better evaluate long-term outcomes; however, the rarity of total colonic aganglionosis remains a significant limitation.

Future research should focus on establishing prospective multicenter registries to increase sample size and improve the quality of evidence for this rare condition. The use of standardized functional outcome measures, such as validated bowel function and quality-of-life scoring systems, would facilitate more reliable comparisons between surgical techniques. In addition, longer-term follow-up extending into adolescence and adulthood is essential to better understand the durability of surgical outcomes, late complications, continence, psychosocial well-being, and overall quality of life.

## Data Availability

The raw data supporting the conclusions of this article will be made available by the authors, without undue reservation.

## References

[1] Hoehner JC Ein SH Shandling B Kim PCW. Long-term morbidity in total colonic aganglionosis. J Pediatr Surg. (1998) 33:961–6. 10.1016/S0022-3468(98)90515-2 9694078

[2] Ieiri S Suita S Nakatsuji T Akiyoshi J Taguchi T. Total colonic aganglionosis with or without small bowel involvement: a 30-year retrospective nationwide survey in Japan. J PediatrSurg. (2008) 43:2226–30. 10.1016/j.jpedsurg.2008.08.049 19040940

[3] Escobar MA Grosfeld JL West KW Scherer LR Rouse TM Engum SA. Long-term outcomes in total colonic aganglionosis: a 32-year experience. J Pediatr Surg. (2005) 40:955–61. 10.1016/j.jpedsurg.2005.03.043 15991177

[4] Bischoff A Levitt MA Peña A. Total colonic aganglionosis: a surgical challenge. How to avoid complications? PediatrSurg Int. (2011) 27:1047–52. 10.1007/s00383-011-2960-y PMC3175048 21809028

[5] Coran AG. A personal experience with 100 consecutive total colectomies and straight ileoanal endorectal pull-throughs for benign disease of the colon and rectum in children and adults. Ann Surg. (1990) 212:242–8. 10.1097/00000658-199009000-00002 2396880 PMC1358148

[6] Kimura K Nishijima E Muraji T Tsugawa C Matsumoto Y. A new surgical approach to extensive aganglionosis. J Pediatr Surg. (1981) 16:840–3. 10.1016/S0022-3468(81)80830-5 7338764

[7] Martin LW. Surgical management of total colonic aganglionosis. Ann Surg. (1972) 176:343–6. 10.1097/00000658-197209000-00010 5073220 PMC1355404

[8] Rintala RJ Lindahl HG. Proctocolectomy and J-pouch ileo-anal anastomosis in children. J Pediatr Surg. (2002) 37:66–70. 10.1053/jpsu.2002.29429 11781989

[9] Marquez TT Acton RD Hess DJ Duval S Saltzman DA. Comprehensive review of procedures for total colonic aganglionosis. J Pediatr Surg. (2009) 44:257–65. 10.1016/j.jpedsurg.2008.10.055 19159753

[10] Bonnard A De Lagausie P Leclair MD. Definitive treatment of extended Hirschsprung’s disease or total colonic form surg. Endosc. (2001) 15:1301–4. 10.1007/s004640090092 11727138

[11] Yagi M Okuyama H Oue T. One-stage laparoscopic pull-through operation without stoma for total colon aganglionosis. Pediatr Endosc Innov Techn. (2003) 7:169–73. 10.1089/109264103766757934

[12] Cheung ST Tam YH Chong HM Chan KW Mou WC Sihoe DY. An 18-year experience in total colonic aganglionosis: from staged operations to primary laparoscopic endorectal pull-through. J Pediatr Surg. (2009) 44(12):2352–4. 10.1016/j.jpedsurg.2009.07.057 20006025

[13] Dodero P Magillo P Scarsi PL. Total colectomy and straight ileo-anal soave endorectal pull-through: personal experience with 42 cases. Eur J Pediatr Surg. (2001) 11(5):319–23. 10.1055/s-2001-18549 11719870

[14] Yeh YT Tsai HL Chen CY Wang JB Chin TW Wei CF. Surgical outcomes of total colonic aganglionosis in children: a 26-year experience in a single institute. J Chin Med Assoc. (2014) 77(10):519–23. 10.1016/j.jcma.2014.05.012 25167835

[15] Ross MN Chang JH Burrington JD Janik JS Wayne ER Clevenger P. Complications of the Martin procedure for total colonic aganglionosis. J Pediatr Surg. (1988) 23(8):725–7. 10.1016/s0022-3468(88)80411-1 3171841

[16] Zhang X Cao GQ Tang ST. Laparoscopic-assisted Duhamel procedure with ex-anal rectal transection for total colonic aganglionosis. J. Pediatr Surg. (2018) 53:531–6. 10.1016/j.jpedsurg.2017.06.009 28705638

[17] Xu WL SL LI Sun C. Single-site laparoscopic-assisted Martin-Duhamel procedure in the treatment of total colonic aganglionosis with stage Ⅰ stoma and evaluation of postoperative defecation function J. Clin Ped Sur. (2020) 19:31–5. 10.3969/j.issn.1671-6353.2020.01.006

[18] Halaweish I Srinivas S Farooqui Z Sutthatarn P Campbell D Frischer J. Duhamel versus Swenson pull-through for total colonic aganglionosis: a multi-institutional study. J Pediatr Surg. (2024) 59(2):216–9. 10.1016/j.jpedsurg.2023.10.017 37973423

[19] Stenström P Kyrklund K Bräutigam M Engstrand Lilja H Juul Stensrud K Löf Granström A. Totalcolonic aganglionosis: multicentre study of surgical treatment and patient-reported outcomes up to adulthood. BJS Open. (2020) 4:943–953. 10.1002/bjs5.50317 PMC7528515 32658386

